# Growth Dynamics of Colloidal Silver–Gold Core–Shell Nanoparticles Studied by *In Situ* Second Harmonic Generation and Extinction Spectroscopy

**DOI:** 10.1021/acs.jpcc.1c06094

**Published:** 2021-11-15

**Authors:** Asela S. Dikkumbura, Prakash Hamal, Min Chen, Daniel A. Babayode, Jeewan C. Ranasinghe, Kenneth Lopata, Louis H. Haber

**Affiliations:** ‡ Department of Chemistry, Louisiana State University, Baton Rouge, Louisiana 70803, United States; † Center for Computation and Technology, Louisiana State University, Baton Rouge, Louisiana 70803, United States

## Abstract

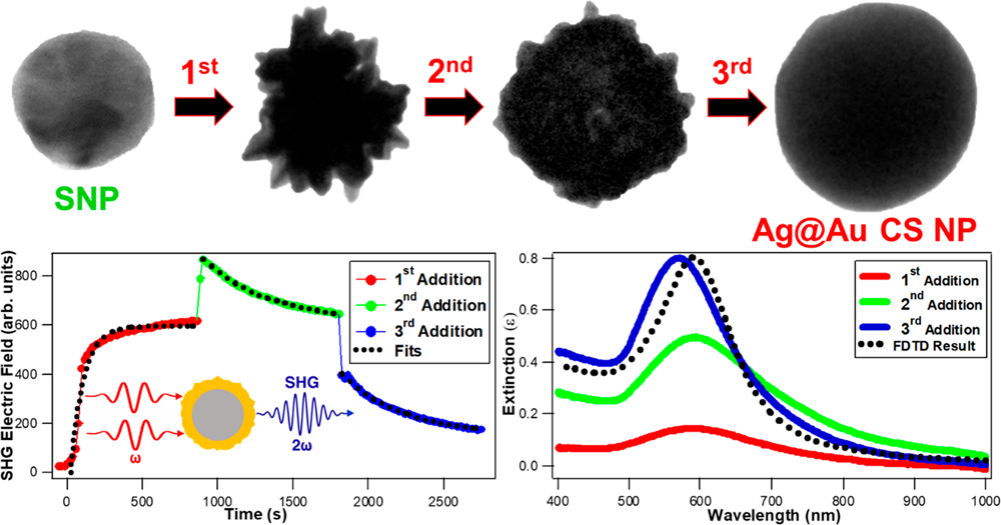

The *in situ* growth dynamics of colloidal silver–gold core–shell (Ag@Au CS) nanoparticles (NPs) in water are monitored in a stepwise synthesis approach using time-dependent second harmonic generation (SHG) and extinction spectroscopy. Three sequential additions of chloroauric acid, sodium citrate, and hydroquinone are added to the silver nanoparticle solution to grow a gold shell around a silver core. The first addition produces a stable urchin-like surface morphology, while the second and third additions continue to grow the gold shell thickness as the surface becomes more smooth and uniform, as determined using transmission electron microscopy. The extinction spectra after each addition are compared to finite-difference time-domain (FDTD) calculations, showing large deviations for the first and second additions due to the bumpy surface morphology and plasmonic hotspots while showing general agreement after the third addition reaches equilibrium. The *in situ* SHG signal is dominated by the NP surface, providing complementary information on the growth time scales due to changes to the surface morphology. This combined approach of synthesis and characterization of Ag@Au CS nanoparticles with *in situ* SHG spectroscopy, extinction spectroscopy, and FDTD calculations provides a detailed foundation for investigating complex colloidal nanoparticle growth mechanisms and dynamics in developing enhanced plasmonic nanomaterial technologies.

## Introduction

Noble metal nanoparticles composed of gold and silver have attracted significant scientific interest due to their unique chemical, electronic, catalytic, and optical properties.^1−4^ These nanomaterials have potential applications in biomedical fields including photothermal cancer therapy, gene therapy, drug delivery, and molecular sensing.^5−8^ The ease of synthesis, biocompatibility, and ability for functionalization with biomolecules through thiol bonds make these nanoparticles advantageous for diagnostic and therapeutic nanomedicines.^9−11^ The optical properties of gold and silver nanoparticles are dominated by tunable, localized surface plasmon resonances, which are characterized by the collective oscillations of free electrons at the nanoparticle surface caused by the incident optical field, leading to enhancement of scattering and absorption processes. The plasmonic spectra depend on the nanoparticle composition, size, shape, surface morphology, and surrounding medium.^12−16^ Hybrid plasmonic nanoparticles composed of gold or silver with spherical core and shell architectures, such as gold–silver core–shell (Au@Ag CS), silver–gold core–shell (Ag@Au CS), and gold–silver–gold core–shell–shell (Au@Ag@Au CSS) nanoparticles provide highly tunable plasmonic spectra.^17−20^ Other types of Ag–Au nanomaterial architectures include Au-protected Ag core/satellite nanoassemblies,^21^ Ag–Au bimetallic nanoalloys,^22,23^ and Ag–Au core–shell nanocubes^24^ for plasmon-based biological applications. Understanding and controlling the size, shape, surface morphology, and associated growth mechanisms of hybrid silver–gold plasmonic nanoparticles is crucial for future developments in nanomaterials, nanoengineering, and nanomedicine.

Numerous methodologies have been studied for preparing bimetallic Ag–Au colloids, Ag–Au alloys, and spherical Ag@Au CS nanoparticles including chemical reduction of chloroauric acid (HAuCl_4_) and silver nitrate (AgNO_3_) in water,^19,20,23,25^ temperature ripening,^26^ laser-induced heating,^27^ laser ablation of bulk alloys,^22^ and microwave-assisted techniques.^28^ Simultaneous reduction of Ag^+^ and AuCl_4_^–^ ions to synthesize Ag–Au bimetallic nanoparticles using plant extracts^29^ and the formation of Ag–Au alloy nanoboxes through a galvanic replacement reaction have also been reported.^30^ Typical solution-based synthesis approaches of Ag@Au CS nanoparticles are based on the reduction of HAuCl_4_ onto the surface of a colloidal silver core using reducing agents such as hydroxylamine hydrochloride,^19,25^ sodium borohydride,^20^ or sodium citrate^23^ (SC) through a seeding growth approach.^31^ Multishell gold–silver nanoparticle synthesis procedures have been reporting where HAuCl_4_ is reduced onto a silver nanoparticle surface using ascorbic acid and a mixture of sodium citrate and hydroquinone (HQ).^17,32^ HQ is a weak reducing agent that facilitates the selective reduction of AuCl_4_^–^ ions onto a colloidal metal nanoparticle surface to form a gold shell while preventing the formation of free gold nanoparticles by secondary nucleation reactions.^33,34^

Many analytical techniques exist for characterizing plasmonic nanoparticles *ex situ* after the synthesis is complete, including UV–vis extinction spectroscopy, dynamic light scattering (DLS), transmission electron microscopy (TEM), atomic force microscopy (AFM), X-ray diffraction (XRD), X-ray photoelectron spectroscopy (XPS), and electron diffraction (ED).^20,25,28,35,36^ However, *in situ* characterization techniques are needed to monitor the real-time growth mechanisms and associated chemical reactions involved in these complex colloidal nanoparticle synthesis procedures for developing improved nanomaterial engineering. For example, *in situ* TEM measurements were used to investigate the growth of Au shells to Pd nanocubes^37^ and Ag shells to Au bipyramidal seeds^38^ in aqueous solution. *In situ* small-angle X-ray scattering was used to study the formation of Ag and Ag–Au alloy nanoparticles under different aqueous colloidal conditions.^39,40^ Additionally, *in situ* second harmonic scattering experiments were used to monitor growth dynamics of Au nanoparticles^41^ and SiO_2_–Au core–shell nanoparticles^42^ in colloidal suspension. In our previous work, *in situ* second harmonic generation (SHG) coupled with extinction spectroscopy was used for real-time monitoring of seed-mediated growth dynamics of colloidal gold nanoparticles and gold–silver core–shell (Au@Ag CS) nanoparticles in water.^43,44^ In our research on gold nanoparticles, enhanced SHG signals were observed at early stages of the growth process due to the formation of plasmonic hot spots from a rough and uneven nanoparticle surface, followed by continued growth that resulted in decreasing SHG signals as the surface became more smooth and uniform.^43^ In our work on Au@Ag CS nanoparticles, the time-dependent *in situ* SHG signals were characterized by biexponential decays where the faster lifetime corresponded to the Ag shell growth onto the Au core, while the slower lifetime was attributed to changes in the nanoparticle surface charge density.^44^ Additionally, the experimental size-dependent Au@Ag CS extinction spectra showed general agreement to theoretically calculated spectra using the finite-difference time-domain (FDTD) approach.^44^ These different *in situ* characterization techniques provide important information on the growth reactions involved in preparing complex hybrid plasmonic nanomaterials.

Second harmonic generation is an interface-selective nonlinear spectroscopic technique that has been used extensively to monitor chemical and structural changes occurring at the surface of colloidal nanoparticles.^45−48^ In SHG, two incident photons of frequency ω combine to produce a photon of frequency 2ω. SHG is dipole-forbidden in centrosymmetric bulk media, such as molecules isotropically distributed in water; however, SHG can be generated from a colloidal nanoparticle surface where the inversion symmetry is broken.^45−52^ Therefore, SHG can be used as a sensitive technique for studying changes in size and surface morphology caused by chemical reactions that take place at the nanoparticle surface during *in situ* nanomaterial synthesis procedures.^41−44,49,51,53,54^ SHG has also been used to investigate the hyperpolarizability of silver nanocubes,^55^ the photothermal release of miRNA nanoparticle conjugates,^48,56−58^ and molecular adsorption and transport at liposome surfaces.^59−61^ Additionally, SHG can provide information on the electrostatic surface potential of nanoparticles from the χ^(3)^ effect.^46,48,49,51,52,62−65^ In our recent work, we used *in situ* SHG and extinction spectroscopy to monitor the seed-mediated growth dynamics of colloidal gold and Au@Ag CS nanoparticles.^43,44^

In this paper, *in situ* SHG spectroscopy coupled with extinction spectroscopy is used to investigate the growth dynamics of colloidal Ag@Au CS nanoparticles in water using a stepwise synthesis procedure. The gold shell is formed onto the colloidal silver core by three sequential additions of HAuCl_4_, sodium citrate, and hydroquinone. The final sizes and surface morphologies of Ag@Au CS NPs after each addition of HAuCl_4_ and reducing agents are determined using TEM, showing urchin-like morphologies after the first addition followed by surface smoothening as the gold shell thickness increases during both the second and third additions. The *in situ* SHG and extinction spectroscopy results are analyzed to determine the associated growth lifetimes for each addition, where the extinction spectra show increasing intensities, blue-shifting, and spectral narrowing as the Ag@Au CS NPs grow in size and where the SHG intensities provide insight on the surface morphology with enhanced signals due to plasmonic hotspots. Corresponding finite-difference time-domain (FDTD) calculations are performed to obtain simulated plasmonic spectra for the Ag@Au CS NPs, showing general agreement with the experimental results after the final addition has reached equilibrium. Investigating the growth dynamics of Ag@Au CS nanoparticles using experimental *in situ* SHG and extinction spectroscopy combined with FDTD computational calculations allows for a detailed understanding and control of the plasmonic nanomaterial size and surface morphology in developing potential hybrid nanoengineering applications.

## Experimental Section

### Nanoparticle Synthesis and Characterization

The synthesis of colloidal Ag@Au CS nanoparticles involves a seed-mediated, stepwise reduction of chloroauric acid with sodium citrate and hydroquinone in three sequential additions. All chemicals are purchased from Alfa Aesar and Sigma-Aldrich and are used without further purification in ultrapure water. First, the colloidal silver nanoparticles are prepared^48,66^ where a 2.60 mL aqueous solution of 5.67 mM silver nitrate, 13.1 mM sodium citrate, and 3.76 μM potassium iodide is added to 47.5 mL of a boiling aqueous solution of 210 μM ascorbic acid. The mixture is refluxed for 60 min under vigorous stirring conditions, where the solution undergoes a color change from colorless to pale greenish yellow as the spherical colloidal silver nanoparticles are formed. Next, HAuCl_4_ is reduced in a stepwise manner onto the silver core to prepare Ag@Au CS nanoparticles through a seed-mediated growth approach.^31^ The size of the gold shell can be controlled by the amount of HAuCl_4_, SC, and HQ added.^17,18^ For the first addition, 15 μL of the prepared silver nanoparticle solution is added to a 2.5 mL aqueous solution of 42.5 μM HAuCl_4_ followed by rapid addition of 11 μL of 7.7 mM SC and 23.2 mM HQ in water to initiate the gold shell growth reaction at the surface of the silver nanoparticles. In the second addition, aqueous solutions of 8.5 μL of 25 mM HAuCl_4_ and 11 μL of 7.7 mM SC and 23.2 mM HQ are added at the same time to the colloidal nanoparticle sample. In the third addition, 12.5 μL of 25 mM HAuCl_4_ and 11 μL of 7.7 mM SC and 23.2 mM HQ are added. These three consecutive additions are done in a quartz cuvette under constant stirring conditions at room temperature while being monitored spectroscopically by *in situ* SHG and extinction spectroscopy with waiting times of 864 s between the first and second additions and 941 s between the second and third additions. TEM images are obtained using a JEOL-1400 microscope with carbon-coated copper grids to find the size distributions and morphologies of the nanoparticles at each step of the synthesis. Additional characterization information on the silver and Ag@Au CS nanoparticles is described in the Supporting Information.

### *In Situ* Second Harmonic Generation and Extinction Spectroscopy

The *in situ* SHG spectroscopy setup has been described previously.^48,50^ Briefly, a titanium:sapphire oscillator laser output centered at 800 nm with 75 fs pulses at a repetition rate of 80 MHz and horizontal polarization is attenuated to 700 mW using a neutral density filter and is focused using a 30 mm focal length lens into a 1 cm quartz cuvette containing the colloidal nanoparticles in aqueous solution. The SHG signal is collected in the forward direction using a 1″ diameter, 50 mm focal length lens and is detected as a function of time using a high-sensitivity spectroscopy charge-coupled device (CCD) connected to a monochromator spectrograph. *In situ* extinction spectra of Ag@Au CS nanoparticles are measured concurrently using a low-intensity broadband beam from a tungsten filament lamp, which is passed through the nanoparticle solution orthogonal to the SHG beam. At time zero, the first addition of SC and HQ reducing agents is added to the solution of Ag NPs and HAuCl_4_ to initiate the gold shell growth process onto the silver nanoparticles, followed by the second and third additions of HAuCl_4_ and reducing agents. Additional details on the *in situ* SHG and extinction spectroscopy setup are provided in the Supporting Information.

### Finite-Difference Time-Domain Calculations

The theoretical extinction spectra of Ag@Au CS nanoparticles with various shell thicknesses are calculated using a classical finite-difference time-domain (FDTD) approach using a custom FDTD code, as described previously.^44,67^ In our calculations, the FDTD approach is used to solve Maxwell’s equations for a single spherical silver–gold core–shell nanoparticle in water using discretized grids in space and time with experimentally fitted spatial- and frequency-dependent permittivities and permeabilities of bulk silver and gold.^67−69^ For each calculation, the grid space is 20 au = 1.06 nm and the time step is chosen to be 0.067 au = 1.62 × 10^–3^ fs such that the Courant–Friedrichs–Lewy stability condition equals to 0.8. The total running time was set as 1500 au = 36.3 fs. The CS nanoparticle geometry for each FDTD calculation was set using a spherical silver core with a spherical gold shell with smooth surfaces. For all calculations, the background was taken to be water, and the system was excited by a discrete Ricker wavelet pulse with a central frequency of 3.20 eV and a width of 0.405 fs.

## Results and Discussion

**Figure 1:**
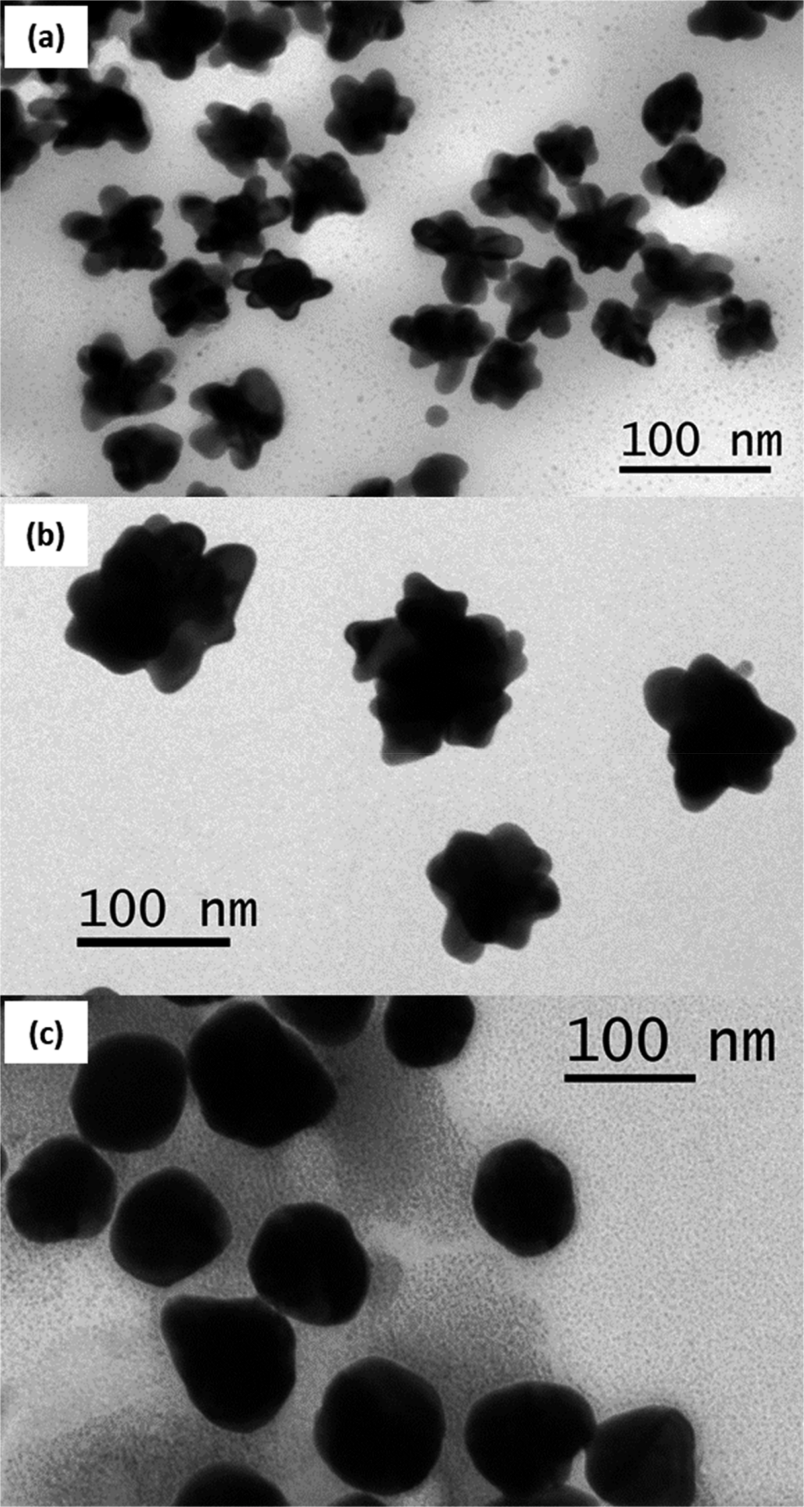
Representative TEM images of Ag@Au CS nanoparticles with average sizes of (a) 56.3 ± 7.6 nm, (b) 94.5 ± 11.8 nm, and (c) 114.7 ± 12.5 nm after the first, second, and third additions of HAuCl_4_ and reducing agents, respectively. These Ag@Au CS nanoparticles all have a silver core diameter of 42.0 ± 6.8 nm.

TEM measurements are used to characterize the Ag@Au CS nanoparticles after each sequential addition of HAuCl_4_ and reducing agents, surveying more than 200 nanoparticles for each sample. The nanoparticle size distribution histograms from the TEM images are fitted to log-normal functions to determine the average nanoparticle diameter, as shown in the Supporting Information. The sizes obtained from the fits are 42.0 ± 6.8 nm for the silver nanoparticles and 56.3 ± 7.6, 94.5 ± 11.8, and 114.7 ± 12.5 nm for the Ag@Au CS nanoparticles after the first, second, and third additions of HAuCl_4_ and reducing agents, respectively. Figure 1 shows representative TEM images of Ag@Au CS nanoparticles after the three sequential additions of HAuCl_4_, SC, and HQ. In the first addition, HAuCl_4_ is reduced onto the silver nanoparticle core, forming urchin-like Ag@Au CS nanoparticles with rough gold surfaces. After the second addition, the nanoparticle size grows larger, and the surface morphology of Ag@Au CS nanoparticles becomes less spiky as compared to the first addition results, but a bumpy, uneven surface is still observed. Finally after the third addition, the Ag@Au CS nanoparticle again increases in size, while the surface morphology becomes much more smooth and uniform. Additional TEM images for the silver and Ag@Au CS NPs are shown in the Supporting Information.

**Figure 2:**
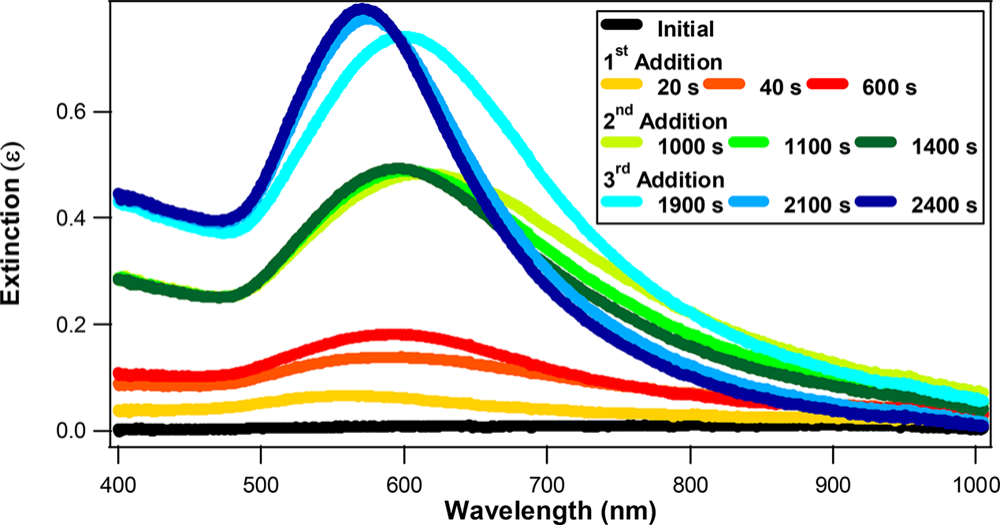
*In situ* extinction spectra of Ag@Au CS nanoparticles during the different stepwise additions of HAuCl_4_ and reducing agents at various reaction times.

*In situ* SHG spectroscopy coupled with extinction spectroscopy is used to investigate the gold shell growth dynamics of the Ag@Au CS nanoparticles. Representative extinction spectra of Ag@Au CS nanoparticles at various times during stepwise sequential additions of HAuCl_4_ and reducing agents are shown in Figure 2. An initial extinction spectrum is obtained before the nanoparticle growth reaction is started, corresponding to the initial solution of colloidal silver nanoparticles and HAuCl_4_ in water. Immediately after the first addition of SC and HQ, a broad plasmon peak centered near 560 nm is observed, which slowly increases in intensity while red-shifting as the nanoparticle growth continues in time, reaching a first equilibrium. These Ag@Au CS nanoparticles at this stage are consistent with “urchin-like”, “blackberry-like”, or “flower-like” morphologies, as shown in Figure 1a, and are characterized by spikey surfaces with plasmonic hot spots and broad, red-shifted plasmonic spectra.^34,70,71^ After the second addition of HAuCl_4_ and reducing agents, the plasmonic peak again rapidly increases in intensity while blue-shifting near 600 nm and narrowing in spectral width. In this stage, the Ag@Au CS nanoparticles continue to grow larger, while the surface roughness decreases until a new equilibrium is reached, as shown in Figure 1b. After the third addition, the plasmonic spectrum again increases rapidly and then approaches a final equilibrium while spectrally narrowing and blue-shifting to a peak near 570 nm. Here, the nanoparticle size again increases, and the surface morphology becomes much more smooth and uniform, as shown in Figure 1c. Additional *in situ* extinction spectra for the three sequential stepwise additions during the Ag@Au CS nanoparticle growth are shown in the Supporting Information.

**Figure 3:**
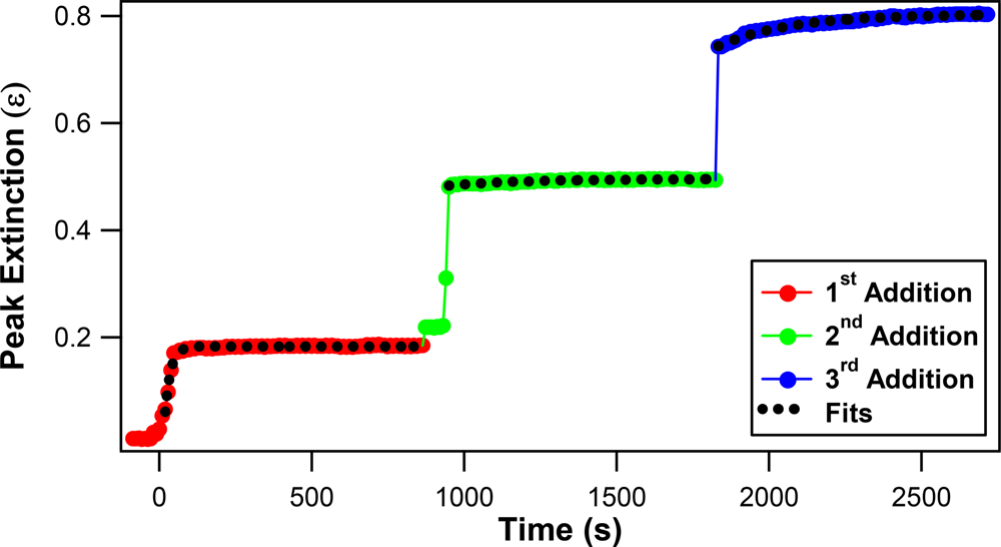
Peak extinction as a function of reaction time during the Ag@Au CS nanoparticle synthesis for the three stepwise additions of HAuCl_4_ and reducing agents, with corresponding fits (black dotted lines).

The *in situ* extinction peak values are displayed as a function of reaction time in Figure 3, with corresponding fits for each stepwise addition. The extinction peak intensities show a very rapid increase immediately after adding HAuCl_4_ and reducing agents, which occurs faster than the current experimental temporal resolution, followed by a slower rise in intensity, reaching an equilibrium for each stepwise addition. The extinction peak values increase for each addition as the overall Ag@Au CS NP size also increases with each addition, as shown by the TEM results. The slower rise components of the extinction peak time traces are fit using a single-exponential function for each addition, given by the equation  to determine the extinction growth lifetimes. Here, *τ*_ext_ is the corresponding extinction growth lifetime, *A*_ext_ is the extinction peak amplitude, and *B*_ext_ is the offset extinction peak value. The measured extinction growth lifetimes are 18 ± 1, 253 ± 24, and 263 ± 11 s for the first, second, and third additions, respectively, and these values are also listed in Table 1. Each of these extinction peak time traces and corresponding fits are also shown separately in the Supporting Information. The corresponding fit parameters for each addition are listed in Table S2 of the Supporting Information. The extinction spectra show no significant deviations during the last 50 s of each stepwise addition, to within experimental signal-to-noise.

**Table 1:** Lifetimes Obtained from the *In Situ* SHG and Extinction Spectroscopy for the Three Additions for the Corresponding Final Sizes in Stepwise Ag@Au CS Nanoparticle Synthesis

addition	final size (nm)	extinction growth lifetime *τ*_ext_ (s)	SHG growth lifetime *τ*_SHG_ (s)
1st	56.3 ± 7.6	18 ± 1	87 ± 6
2nd	94.5 ± 11.8	253 ± 24	439 ± 6
3rd	114.7 ± 12.5	263 ± 11	409 ± 13

**Figure 4:**
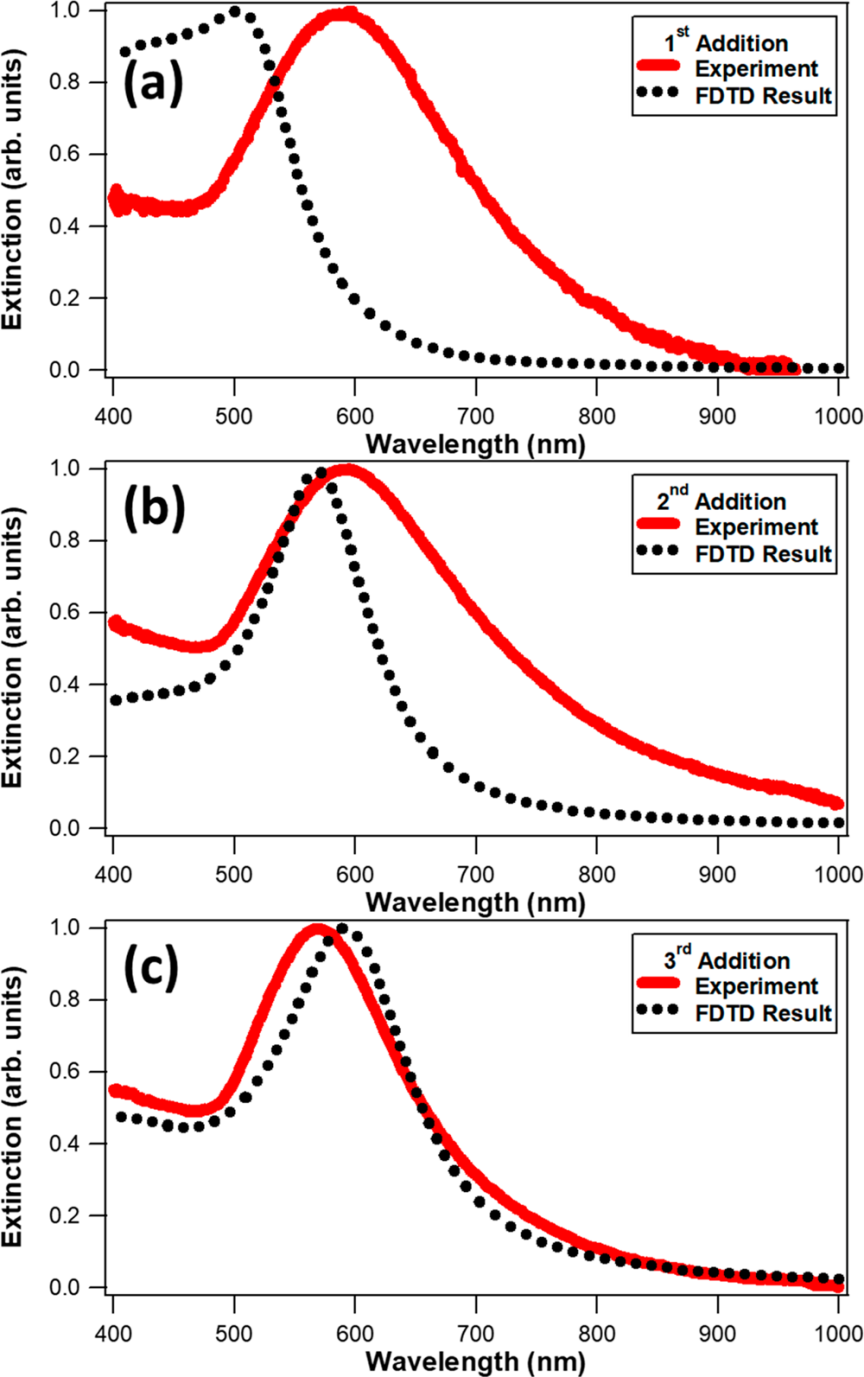
Normalized final extinction spectra (red lines) of Ag@Au CS nanoparticles after the (a) first, (b) second, and (c) third additions of HAuCl_4_ and reducing agents, respectively, with corresponding FDTD results (dotted lines). Large deviations between the experiment and theory after the first and second additions are due primarily to the urchin-like surface morphologies. General agreement after the third addition demonstrates a smoother final CS structure.

The final extinction spectra of Ag@Au CS nanoparticles after each of the three stepwise additions of HAuCl_4_ and reducing agents are compared with corresponding FDTD calculations, as shown in Figure 4. The final extinction spectra correspond to reaction times of 864, 1804, and 2745 s, which are at the end of the first, second, and third additions, respectively, when the extinction spectra are stable. The FDTD spectra are calculated by assuming an ideal architecture composed of a perfect spherical silver core diameter of 42.0 nm and a uniform spherical gold shell corresponding to the average sizes measured by TEM, with gold shell thicknesses of 7.15, 26.25, and 30.10 nm for the first, second, and third additions, respectively. These calculated spectra are the sum of absorption and scattering,^67^ providing a direct comparison to the experimental results. The FDTD-calculated spectra have peaks at 501, 569, and 595 nm respectively for the ideal Ag@Au CS nanoparticles corresponding to the average sizes after the first, second, and third additions. These results compare to the final experimental extinction spectra for the three additions, after reaching each corresponding equilibrium, with peak wavelengths of 597, 588, and 571 nm, for the first, second, and third additions, respectively. The experimental results show significantly red-shifted spectra after the first and second additions caused by deviations from ideal core–shell nanoarchitectures due to the surface morphology and plasmonic hotspots. The full widths at half maxima (fwhm) from the final experimental extinction spectra are 216, 245, and 177 nm for three sequential additions, respectively, and 215, 108, and 149 nm for the corresponding FDTD-calculated spectra, respectively. The experimental spectrum after the final addition shows a general agreement with the FDTD-calculated spectrum, both in peak wavelength and fwhm, although some discrepancies remain, which we attribute to some degree of polydispersity in Ag@Au CS nanoparticle shape, surface unevenness, and roughness at the silver–gold core–shell interface after the third addition of the stepwise synthesis procedure is complete.

**Figure 5:**
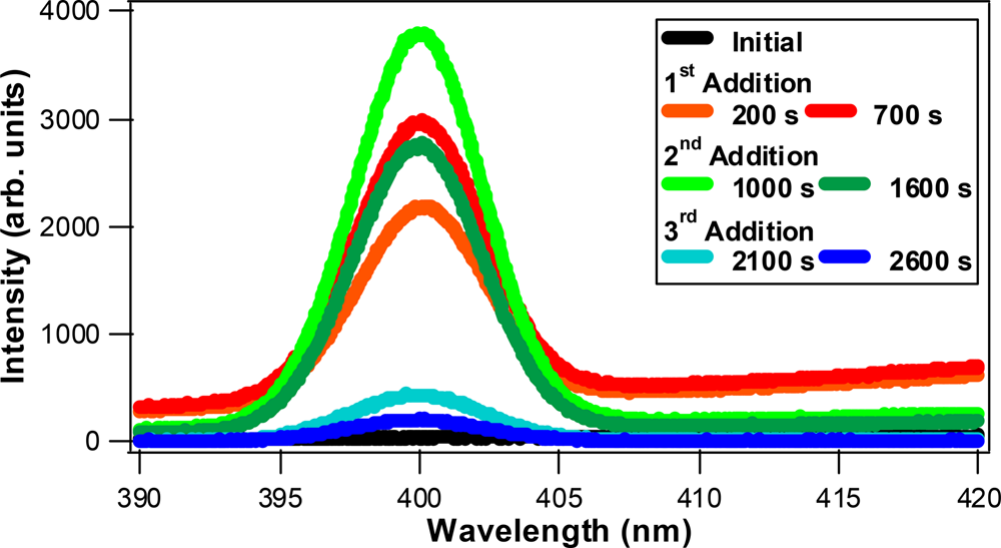
*In situ* SHG spectra of Ag@Au CS nanoparticles during the different stepwise additions of HAuCl_4_ and reducing agents at various reaction times.

The *in situ* SHG measurements give important complementary information for understanding the colloidal nanoparticle growth mechanisms due to the surface sensitivity of the technique.^42−44^ Representative *in situ* SHG spectra during the formation of Ag@Au CS nanoparticles at selected reaction times are shown in Figure 5. The initial reaction mixture contains silver nanoparticles and HAuCl_4_ in ultrapure water and gives very low SHG signal. During the first addition, the SHG signal rapidly increases and reaches a first equilibrium of stable SHG signal over time. The SHG peak is centered at 400 nm with a fwhm of 6 nm. A very broad signal of two-photon fluorescence (TPF)^45,48^ that appears as an upward sloping baseline is also observed in this stage of the Ag@Au CS nanoparticle growth reaction. For the second addition, the SHG intensity first increases abruptly upon adding HAuCl_4_ and reducing agents and then decreases gradually during this stage of the growth process until reaching a second equilibrium. For the final addition, the SHG intensity first rapidly decreases then continues to decrease more gradually until reaching a final equilibrium. As described in our previous work studying *in situ* Au and Au@Ag CS NP growth dynamics, the SHG signal depends on both the nanoparticle size as well as the surface morphology from plasmonic hotspots.^43,44,72,73^ The SHG signal can also depend on the nanoparticle composition, shape, and surrounding medium as well as the optical polarization configuration and scattering angles used.^46,47,74^ The TPF signals in our measurements decrease substantially during the Ag@Au CS nanoparticle growth reactions in the second and third additions, indicating that the relative TPF signal becomes enhanced from plasmonic hot spots in the first addition and then decreases as the surface becomes more smooth in the second and third additions.^75^

**Figure 6:**
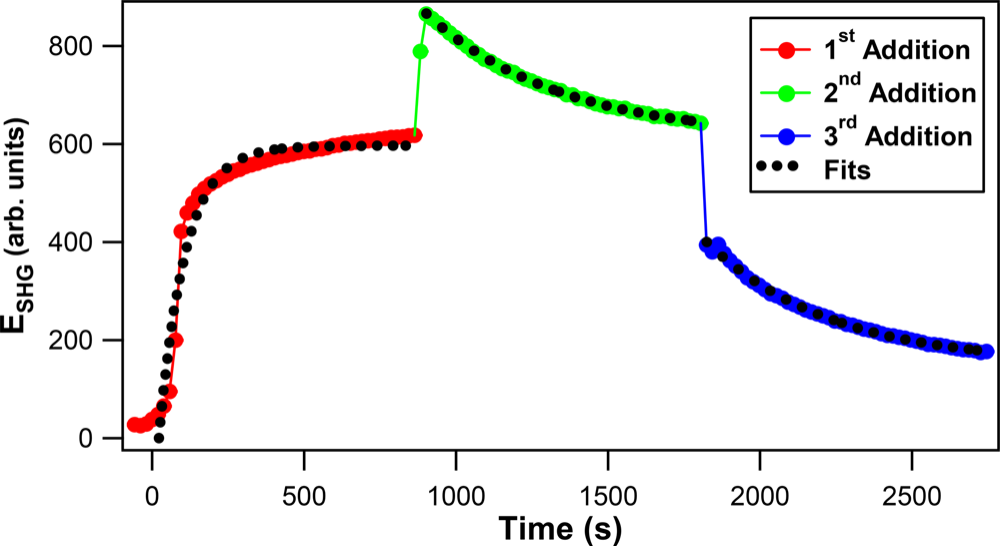
SHG electric field during the first (red circles), second (green circles), and third (blue circles) additions of HAuCl_4_ and reducing agents as a function of reaction time with corresponding single-exponential fits (black dotted lines) in the stepwise synthesis of Ag@Au CS nanoparticles.

The time trace of the SHG electric field during the gold shell formation onto the colloidal silver core is shown in Figure 6 for the three sequential additions of HAuCl_4_ and reducing agents. The corrected SHG signal intensity *I*_SHG,Corr_ for Ag@Au CS nanoparticles is obtained to account for the time-dependent linear extinction response using the equation^42,44^, where *I*_SHG,Expt_, ε_800_, and ε_400_ are the experimentally measured SHG intensity and the extinctions obtained from spectra of Ag@Au CS nanoparticles at 800 and 400 nm, respectively. The corrected SHG electric field *E*_SHG_ is calculated from the square root of the integrated corrected SHG signal where . After the first addition, the SHG intensity steadily increases, reaching a plateau equilibrium as the nanoparticle size increases with significant urchin-like surface roughness and corresponding plasmonic hotspots that produce enhanced SHG signals. After the second addition, a rapid increase in the SHG electric field is observed followed by a more gradual decay caused by the surface becoming more smooth as the Ag@Au CS nanoparticles grow in size. With the third addition, a sharp decrease in SHG electric field is observed followed again by a gradual decay as the CS nanoparticle surface morphology becomes much more smooth and uniform with a corresponding decrease in plasmonic hotspots, eventually reaching the final equilibrium of a more ideal spherical core–shell nanoarchitecture.

The SHG electric field time trace for each stepwise addition is fit using a single-exponential function given by the equation  to determine the corresponding SHG growth lifetimes. Here, *t* is the reaction time after the addition of the reducing agents, *τ*_SHG_ is the SHG growth lifetime, *A*_SHG_ is the SHG amplitude, and *B*_SHG_ is the offset SHG electric field. The fits for each addition are shown in Figure 6 as dotted black lines, and the SHG growth lifetimes are determined to be 87 ± 6, 439 ± 6, and 409 ± 13 s for the first, second, and third additions, respectively. These SHG growth lifetimes are listed in Table 1, and the fitting parameters are also listed in Table S1 of the Supporting Information. The SHG signal arises predominantly from the nonlocal excitation of the electric-dipole moment and the local excitation of the electric-quadrupole moment, where polarization-dependent and angular scattering-dependent measurements are needed for determining these relative contributions in comparison to theoretical modeling.^47,74,76,77^

A comparison between the extinction growth lifetimes and the SHG growth lifetimes for the different stepwise additions provides more insight into the Ag@Au CS nanoparticle growth dynamics. The extinction growth lifetimes are dominated by the bulk nanomaterials, while the SHG growth lifetimes are dominated more by the surface due to the different spectroscopic processes. In the second and third additions, the extinction growth lifetimes are both similar at 253 and 263 s, respectively, while the SHG growth lifetimes are also similar at 439 and 409 s, respectively. This indicates that the overall shell thickness reaches an equilibrium faster, while the surface morphology takes longer to reach its equilibrium structure for these additions. In the first addition, both the extinction and SHG growth lifetimes are much faster at 18 and 87 s, respectively. However, the ratio of *τ*_SHG_/*τ*_ext_ is larger in the first addition at 4.8 ± 0.4 compared to 1.7 ± 0.2 and 1.6 ± 0.1 for the second and third additions, respectively. This highlights the different processes of gold growing on a silver surface in the first addition compared to gold growing on a gold surface in the second and third additions. Additionally, gold growing on silver results in a very spikey urchin-like morphology characterized by increasing SHG over time due to large plasmonic hotspots. Alternatively, gold growing on gold results in a surface morphology becoming more smooth over time as the SHG signal decreases.

Additionally, comparisons to our previous studies on *in situ* SHG and extinction spectroscopy of growth dynamics of seed-mediated Au nanoparticles^43^ and Au@Ag CS nanoparticles^44^ allow for overall trends to be identified in these different plasmonic nanoparticle synthesis approaches. In seed-mediated gold nanoparticle growth using SC and HQ reduction of HAuCl_4_ onto a gold surface, rapid growth is first observed with plasmonic hotspots followed by slower growth with the surface becoming more smooth over time, resulting in a uniform, spherical shape with each addition.^43^ For the seed-mediated Au@Ag CS nanoparticle growth using ascorbic acid reduction of AgNO_3_ onto a gold surface, rapid growth is again observed followed by fast growth lifetimes with surface smoothening and a second slower lifetime caused by changes in the surface charge density. Here, silver grows on gold faster with less plasmonic hotspots than in gold growing on gold. In comparison, gold growing on silver, as studied here with Ag@Au CS nanoparticles, shows significant plasmonic hotspots with stable, urchin-like surface morphologies after the first addition. The gold shell surface becomes smooth only after multiple additions of reducing agents and HAuCl_4_, where the surface morphology reaches its equilibrium structure on a longer time scale than the shell thickness growth. Plasmonic hotspots in the early stages of Ag@Au CS synthesis can be advantageous for certain applications such as in molecular sensing and photothermal therapies.^20,21^ Additionally, gold nanoshells growing on a silver core show no clear changes in surface charge density from the χ^(3)^ effect.^44,52,64^ Future work on the growth dynamics of Ag@Au CS nanoparticles will include different reaction conditions, surface characterizations, and final sizes for a more extensive study. By using *in situ* SHG and extinction spectroscopy, combined with additional characterization tools such as FDTD modeling and TEM, a wide variety of nanomaterial growth dynamics can be investigated, including studies of more sophisticated structures with more than two metals or materials, multiple layered shells, and multifunctional particles.^17,57^ Additionally, different types of plasmonic core–shell nanoparticle architectures and their associated growth dynamics can be used for developing new plasmonic nanomaterials that are specially tailored for specific nanomedicine, catalytic, and optoelectronic technologies.

## Conclusions

The growth dynamics of silver–gold core–shell nanoparticles in a colloidal, seed-mediated, stepwise synthesis are studied using *in situ* SHG and extinction spectroscopy combined with FDTD calculations. The synthesis procedure is based on three sequential additions of chloroauric acid and the reducing agents sodium citrate and hydroquinone to grow spherical gold shells onto 42 nm spherical silver nanoparticles in aqueous suspension. A stable, urchin-like Ag@Au CS surface morphology is observed after the first addition reaches equilibrium, with an average size of 56 nm as determined by TEM measurements. The Ag@Au CS nanoparticle surface becomes more smooth and uniform after the second and third additions, reaching a final size of 115 nm. The *in situ* extinction spectra show increases in intensity for each addition, with blue-shifting and spectral narrowing as the nanoparticles grow in size. Simulated plasmonic spectra calculated using the finite-difference time-domain approach give general agreement with the experimental extinction spectrum of the Ag@Au CS nanoparticle after the third addition of the stepwise synthesis procedure is complete. The *in situ* time-dependent SHG results are dominated by changes to the surface morphology, showing abrupt changes immediately after the additions of HAuCl_4_ and the reducing agents, followed by a gradual exponential increase as a function of time during the first addition and gradual exponential decreases during the second and third additions. The corresponding growth lifetimes from the *in situ* SHG and extinction spectroscopy results highlight the different surface and bulk sensitivities of these optical techniques, providing important insight for controlling enhanced plasmonic nanoengineering applications.

## Supporting Information Available

Additional TEM images of silver and Ag@Au CS nanoparticles with their corresponding size distributions, the *in situ* SHG and extinction setup, peak extinction time profiles, and tabulated fitting parameters (PDF)

## References

[ref1] Link S.; El-Sayed M. A. Optical properties and ultrafast dynamics of metallic nanocrystals. Annu. Rev. Phys. Chem. 2003, 54, 331–366. 10.1146/annurev.physchem.54.011002.103759. 12626731

[ref2] Hu M.; Chen J.; Li Z.-Y.; Au L.; Hartland G. V.; Li X.; Marquez M.; Xia Y. Gold nanostructures: engineering their plasmonic properties for biomedical applications. Chem. Soc. Rev. 2006, 35 (11), 1084–1094. 10.1039/b517615h. 17057837

[ref3] Boken J.; Khurana P.; Thatai S.; Kumar D.; Prasad S. Plasmonic nanoparticles and their analytical applications: A review. Appl. Spectrosc. Rev. 2017, 52 (9), 774–820. 10.1080/05704928.2017.1312427.

[ref4] Kamat P. V. Photophysical, photochemical and photocatalytic aspects of metal nanoparticles. J. Phys. Chem. B 2002, 106 (32), 7729–7744. 10.1021/jp0209289.

[ref5] Lal S.; Clare S. E.; Halas N. J. Nanoshell-enabled photothermal cancer therapy: impending clinical impact. Acc. Chem. Res. 2008, 41, 1842–1851. 10.1021/ar800150g. 19053240

[ref6] Knipe J. M.; Peters J. T.; Peppas N. A. Theranostic agents for intracellular gene delivery with spatiotemporal imaging. Nano Today 2013, 8, 21–38. 10.1016/j.nantod.2012.12.004. 23606894 PMC3627379

[ref7] Song J.; Hwang S.; Im K.; Hur J.; Nam J.; Hwang S.; Ahn G.-O.; Kim S.; Park N. Light-responsible DNA hydrogel-gold nanoparticle assembly for synergistic cancer therapy. J. Mater. Chem. B 2015, 3, 1537–1543. 10.1039/C4TB01519C. 32262426

[ref8] Zhao X.; Hu W.; Wang Y.; Zhu L.; Yang L.; Sha Z.; Zhang J. Decoration of graphene with 2-aminoethanethiol functionalized gold nanoparticles for molecular imprinted sensing of erythrosine. Carbon 2018, 127, 618–626. 10.1016/j.carbon.2017.11.041.

[ref9] Thakor A. S.; Gambhir S. S. Nanooncology: the future of cancer diagnosis and therapy. Ca-Cancer J. Clin. 2013, 63, 395–418. 10.3322/caac.21199. 24114523

[ref10] Dreaden E. C.; Alkilany A. M.; Huang X.; Murphy C. J.; El-Sayed M. A. The golden age: gold nanoparticles for biomedicine. Chem. Soc. Rev. 2012, 41, 2740–2779. 10.1039/C1CS15237H. 22109657 PMC5876014

[ref11] Couvreur P. Nanoparticles in drug delivery: past, present and future. Adv. Drug Delivery Rev. 2013, 65, 21–23. 10.1016/j.addr.2012.04.010. 22580334

[ref12] Thomas K. G.; Kamat P. V. Chromophore-functionalized gold nanoparticles. Acc. Chem. Res. 2003, 36, 888–898. 10.1021/ar030030h. 14674780

[ref13] Jain P. K.; Lee K. S.; El-Sayed I. H.; El-Sayed M. A. Calculated absorption and scattering properties of gold nanoparticles of different size, shape, and composition: applications in biological imaging and biomedicine. J. Phys. Chem. B 2006, 110, 7238–7248. 10.1021/jp057170o. 16599493

[ref14] Xia Y.; Xiong Y.; Lim B.; Skrabalak S. E. Shape controlled synthesis of metal nanocrystals: simple chemistry meets complex physics?. Angew. Chem., Int. Ed. 2009, 48, 60–103. 10.1002/anie.200802248. PMC2791829 19053095

[ref15] Willets K. A.; Van Duyne R. P. Localized surface plasmon resonance spectroscopy and sensing. Annu. Rev. Phys. Chem. 2007, 58, 267–297. 10.1146/annurev.physchem.58.032806.104607. 17067281

[ref16] Sherry L. J.; Chang S.-H.; Schatz G. C.; Van Duyne R. P.; Wiley B. J.; Xia Y. Localized surface plasmon resonance spectroscopy of single silver nanocubes. Nano Lett. 2005, 5, 2034–2038. 10.1021/nl0515753. 16218733

[ref17] Karam T. E.; Smith H. T.; Haber L. H. Enhanced photothermal effects and excited-state dynamics of plasmonic size-controlled gold-silver-gold core-shell-shell nanoparticles. J. Phys. Chem. C 2015, 119, 18573–18580. 10.1021/acs.jpcc.5b05110.

[ref18] Samal A. K.; Polavarapu L.; Rodal-Cedeira S.; Liz-Marzán L. M.; Pérez-Juste J.; Pastoriza-Santos I. Size tunable Au@ Ag core-shell nanoparticles: synthesis and surface-enhanced Raman scattering properties. Langmuir 2013, 29, 15076–15082. 10.1021/la403707j. 24261458

[ref19] Guo X.; Guo Z.; Jin Y.; Liu Z.; Zhang W.; Huang D. Silver-gold core-shell nanoparticles containing methylene blue as SERS labels for probing and imaging of live cells. Microchim. Acta 2012, 178, 229–236. 10.1007/s00604-012-0829-y.

[ref20] Cao Y.; Jin R.; Mirkin C. A. DNA-modified core- shell Ag/Au nanoparticles. J. Am. Chem. Soc. 2001, 123, 7961–7962. 10.1021/ja011342n. 11493092

[ref21] Zhang Z.; Bando K.; Taguchi A.; Mochizuki K.; Sato K.; Yasuda H.; Fujita K.; Kawata S. Au-Protected Ag Core/Satellite Nanoassemblies for Excellent Extra-/Intracellular Surface-Enhanced Raman Scattering Activity. ACS Appl. Mater. Interfaces 2017, 9, 44027–44037. 10.1021/acsami.7b14976. 29171749

[ref22] Lee I.; Han S. W.; Kim K. Production of Au-Ag alloy nanoparticles by laser ablation of bulk alloys. Chem. Commun. 2001, 1782–1783. 10.1039/b105437f. 12240313

[ref23] Csapó E.; Oszkó A.; Varga E.; Juhász Á.; Buzás N.; Kőrösi L.; Majzik A.; Dékány I. Synthesis and characterization of Ag/Au alloy and core (Ag)-shell (Au) nanoparticles. Colloids Surf., A 2012, 415, 281–287. 10.1016/j.colsurfa.2012.09.005.

[ref24] Yang Y.; Liu J.; Fu Z.-W.; Qin D. Galvanic replacement-free deposition of Au on Ag for core-shell nanocubes with enhanced chemical stability and SERS activity. J. Am. Chem. Soc. 2014, 136, 8153–8156. 10.1021/ja502472x. 24863686

[ref25] Srnová-Šloufová I.; Lednický F.; Gemperle A.; Gemperlová J. Core- shell (Ag) Au bimetallic nanoparticles: Analysis of transmission electron microscopy images. Langmuir 2000, 16, 9928–9935. 10.1021/la0009588.

[ref26] Ji Y.; Yang S.; Guo S.; Song X.; Ding B.; Yang Z. Bimetallic Ag/Au nanoparticles: A low temperature ripening strategy in aqueous solution. Colloids Surf., A 2010, 372, 204–209. 10.1016/j.colsurfa.2010.10.028.

[ref27] Povolotskiy A.; Povolotckaia A.; Petrov Y.; Manshina A.; Tunik S. Laser-induced synthesis of metallic silver-gold nanoparticles encapsulated in carbon nanospheres for surface-enhanced Raman spectroscopy and toxins detection. Appl. Phys. Lett. 2013, 103, 113102 10.1063/1.4820841.

[ref28] El-Naggar M. E.; Shaheen T. I.; Fouda M. M.; Hebeish A. A. Eco-friendly microwave-assisted green and rapid synthesis of well-stabilized gold and core-shell silver-gold nanoparticles. Carbohydr. Polym. 2016, 136, 1128–1136. 10.1016/j.carbpol.2015.10.003. 26572455

[ref29] Shankar S. S.; Rai A.; Ahmad A.; Sastry M. Rapid synthesis of Au, Ag, and bimetallic Au core-Ag shell nanoparticles using Neem (Azadirachta indica) leaf broth. J. Colloid Interface Sci. 2004, 275, 496–502. 10.1016/j.jcis.2004.03.003. 15178278

[ref30] Lin G.; Dong W.; Wang C.; Lu W. Mechanistic study on galvanic replacement reaction and synthesis of Ag-Au alloy nanoboxes with good surface-enhanced Raman scattering activity to detect melamine. Sens. Actuators, B 2018, 263, 274–280. 10.1016/j.snb.2018.02.112.

[ref31] Turkevich J.; Kim G. Palladium: preparation and catalytic properties of particles of uniform size. Science 1970, 169, 873–879. 10.1126/science.169.3948.873. 17750062

[ref32] Knauer A.; Thete A.; Li S.; Romanus H.; Csaki A.; Fritzsche W.; Köhler J. Au/Ag/Au double shell nanoparticles with narrow size distribution obtained by continuous micro segmented flow synthesis. Chem. Eng. J. 2011, 166, 1164–1169. 10.1016/j.cej.2010.12.028.

[ref33] Perrault S. D.; Chan W. C. Synthesis and surface modification of highly monodispersed, spherical gold nanoparticles of 50- 200 nm. J. Am. Chem. Soc. 2009, 131, 17042–17043. 10.1021/ja907069u. 19891442

[ref34] Li J.; Wu J.; Zhang X.; Liu Y.; Zhou D.; Sun H.; Zhang H.; Yang B. Controllable synthesis of stable urchin-like gold nanoparticles using hydroquinone to tune the reactivity of gold chloride. J. Phys. Chem. C 2011, 115, 3630–3637. 10.1021/jp1119074.

[ref35] Mourdikoudis S.; Pallares R. M.; Thanh N. T. Characterization techniques for nanoparticles: comparison and complementarity upon studying nanoparticle properties. Nanoscale 2018, 10, 12871–12934. 10.1039/C8NR02278J. 29926865

[ref36] Liz-Marzán L. M. Tailoring surface plasmons through the morphology and assembly of metal nanoparticles. Langmuir 2006, 22 (1), 32–41. 10.1021/la0513353. 16378396

[ref37] Su T.; Wang Z. L.; Wang Z. In Situ Observations of Shell Growth and Oxidative Etching Behaviors of Pd Nanoparticles in Solutions by Liquid Cell Transmission Electron Microscopy. Small 2019, 15, 1900050 10.1002/smll.201900050. 30844138

[ref38] Aliyah K.; Lyu J.; Goldmann C.; Bizien T.; Hamon C.; Alloyeau D.; Constantin D. Real-Time In Situ Observations Reveal a Double Role for Ascorbic Acid in the Anisotropic Growth of Silver on Gold. J. Phys. Chem. Lett. 2020, 11 (8), 2830–2837. 10.1021/acs.jpclett.0c00121. 32200632

[ref39] Garcia P. R.; Prymak O.; Grasmik V.; Pappert K.; Wlysses W.; Otubo L.; Epple M.; Oliveira C. L. An in situ SAXS investigation of the formation of silver nanoparticles and bimetallic silver-gold nanoparticles in controlled wet-chemical reduction synthesis. Nanoscale Adv. 2020, 2, 225–238. 10.1039/C9NA00569B. PMC9418936 36133991

[ref40] Polte J.; Tuaev X.; Wuithschick M.; Fischer A.; Thuenemann A. F.; Rademann K.; Kraehnert R.; Emmerling F. Formation Mechanism of Colloidal Silver Nanoparticles: Analogies and Differences to the Growth of Gold Nanoparticles. ACS Nano 2012, 6 (7), 5791–5802. 10.1021/nn301724z. 22681612

[ref41] de Coene Y.; Deschaume O.; Jooken S.; Seré S.; Van Cleuvenbergen S.; Bartic C.; Verbiest T.; Clays K. Advent of Plasmonic Behavior: Dynamically Tracking the Formation of Gold Nanoparticles through Nonlinear Spectroscopy. Chem. Mater. 2020, 32 (17), 7327–7337. 10.1021/acs.chemmater.0c02178.

[ref42] Sauerbeck C.; Haderlein M.; Schürer B.; Braunschweig B. r.; Peukert W.; Klupp Taylor R. N. Shedding light on the growth of gold nanoshells. ACS Nano 2014, 8, 3088–3096. 10.1021/nn500729r. 24552660

[ref43] Khoury R. A.; Ranasinghe J. C.; Dikkumbura A. S.; Hamal P.; Kumal R. R.; Karam T. E.; Smith H. T.; Haber L. H. Monitoring the Seed-Mediated Growth of Gold Nanoparticles Using in Situ Second Harmonic Generation and Extinction Spectroscopy. J. Phys. Chem. C 2018, 122, 24400–24406. 10.1021/acs.jpcc.8b07176.

[ref44] Ranasinghe J. C.; Dikkumbura A. S.; Hamal P.; Chen M.; Khoury R. A.; Smith H. T.; Lopata K.; Haber L. H. Monitoring the growth dynamics of colloidal gold-silver core-shell nanoparticles using in situ second harmonic generation and extinction spectroscopy. J. Chem. Phys. 2019, 151, 224701 10.1063/1.5127941. 31837661

[ref45] Haber L. H.; Kwok S. J.; Semeraro M.; Eisenthal K. B. Probing the colloidal gold nanoparticle/aqueous interface with second harmonic generation. Chem. Phys. Lett. 2011, 507, 11–14. 10.1016/j.cplett.2011.03.042.

[ref46] Eisenthal K. B. Second harmonic spectroscopy of aqueous nano-and microparticle interfaces. Chem. Rev. 2006, 106, 1462–1477. 10.1021/cr0403685. 16608187

[ref47] Roke S.; Gonella G. Nonlinear light scattering and spectroscopy of particles and droplets in liquids. Annu. Rev. Phys. Chem. 2012, 63, 353–378. 10.1146/annurev-physchem-032511-143748. 22263911

[ref48] Kumal R. R.; Abu-Laban M.; Landry C. R.; Kruger B.; Zhang Z.; Hayes D. J.; Haber L. H. Plasmon-enhanced photocleaving dynamics in colloidal microRNA-functionalized silver nanoparticles monitored with second harmonic generation. Langmuir 2016, 32, 10394–10401. 10.1021/acs.langmuir.6b02538. 27605308 PMC5124014

[ref49] Hayes P. L.; Malin J. N.; Jordan D. S.; Geiger F. M. Get charged up: Nonlinear optical voltammetry for quantifying the thermodynamics and electrostatics of metal cations at aqueous/oxide interfaces. Chem. Phys. Lett. 2010, 499, 183–192. 10.1016/j.cplett.2010.09.060.

[ref50] Karam T. E.; Haber L. H. Molecular adsorption and resonance coupling at the colloidal gold nanoparticle interface. J. Phys. Chem. C 2014, 118, 642–649. 10.1021/jp410128v.

[ref51] Yan E. C.; Liu Y.; Eisenthal K. B. New method for determination of surface potential of microscopic particles by second harmonic generation. J. Phys. Chem. B 1998, 102, 6331–6336. 10.1021/jp981335u.

[ref52] Kumal R. R.; Karam T. E.; Haber L. H. Determination of the surface charge density of colloidal gold nanoparticles using second harmonic generation. J. Phys. Chem. C 2015, 119 (28), 16200–16207. 10.1021/acs.jpcc.5b00568.

[ref53] Zhao X.; Ong S.; Wang H.; Eisenthal K. B. New method for determination of surface pKa using second harmonic generation. Chem. Phys. Lett. 1993, 214, 203–207. 10.1016/0009-2614(93)90082-C.

[ref54] Valev V. Characterization of nanostructured plasmonic surfaces with second harmonic generation. Langmuir 2012, 28, 15454–15471. 10.1021/la302485c. 22889193

[ref55] Russier-Antoine I.; Lee H. J.; Wark A. W.; Butet J.; Benichou E.; Jonin C.; Martin O. J.; Brevet P.-F. Second harmonic scattering from silver nanocubes. J. Phys. Chem. C 2018, 122, 17447–17455. 10.1021/acs.jpcc.8b04299.

[ref56] Abu Laban M.; Hamal P.; Arrizabalaga J. H.; Forghani A.; Dikkumbura A. S.; Kumal R. R.; Haber L. H.; Hayes D. J. Combinatorial Delivery of miRNA Nanoparticle Conjugates in Human Adipose Stem Cells for Amplified Osteogenesis. Small 2019, 15, 1902864 10.1002/smll.201902864. PMC8530457 31725198

[ref57] Kumal R. R.; Abu-Laban M.; Hamal P.; Kruger B.; Smith H. T.; Hayes D. J.; Haber L. H. Near-Infrared Photothermal Release of siRNA from the Surface of Colloidal Gold-Silver-Gold Core-Shell-Shell Nanoparticles Studied with Second-Harmonic Generation. J. Phys. Chem. C 2018, 122 (34), 19699–19704. 10.1021/acs.jpcc.8b06117. PMC6326572 30637038

[ref58] Kumal R. R.; Landry C. R.; Abu-Laban M.; Hayes D. J.; Haber L. H. Monitoring the Photocleaving Dynamics of Colloidal MicroRNA-Functionalized Gold Nanoparticles Using Second Harmonic Generation. Langmuir 2015, 31 (36), 9983–9990. 10.1021/acs.langmuir.5b02199. 26313536 PMC4819427

[ref59] Kumal R. R.; Nguyenhuu H.; Winter J. E.; McCarley R. L.; Haber L. H. Impacts of Salt, Buffer, and Lipid Nature on Molecular Adsorption and Transport in Liposomes As Observed by Second Harmonic Generation. J. Phys. Chem. C 2017, 121 (29), 15851–15860. 10.1021/acs.jpcc.7b05058.

[ref60] Hamal P.; Nguyenhuu H.; Subasinghege Don V.; Kumal R. R.; Kumar R.; McCarley R. L.; Haber L. H. Molecular Adsorption and Transport at Liposome Surfaces Studied by Molecular Dynamics Simulations and Second Harmonic Generation Spectroscopy. J. Phys. Chem. B 2019, 123, 7722–7730. 10.1021/acs.jpcb.9b05954. 31407578

[ref61] Wilhelm M. J.; Sharifian Gh M.; Dai H. L. Influence of molecular structure on passive membrane transport: A case study by second harmonic light scattering. J. Chem. Phys. 2019, 150 (10), 104705 10.1063/1.5081720. 30876365

[ref62] Campen R. K.; Pymer A. K.; Nihonyanagi S.; Borguet E. Linking Surface Potential and Deprotonation in Nanoporous Silica: Second Harmonic Generation and Acid/Base Titration. J. Phys. Chem. C 2010, 114 (43), 18465–18473. 10.1021/jp1037574.

[ref63] Rehl B.; Rashwan M.; DeWalt-Kerian E. L.; Jarisz T. A.; Darlington A. M.; Hore D. K.; Gibbs J. M. New Insights into χ(3) Measurements: Comparing Nonresonant Second Harmonic Generation and Resonant Sum Frequency Generation at the Silica/Aqueous Electrolyte Interface. J. Phys. Chem. C 2019, 123 (17), 10991–11000. 10.1021/acs.jpcc.9b01300.

[ref64] Ohno P. E.; Chang H.; Spencer A. P.; Liu Y.; Boamah M. D.; Wang H.-f.; Geiger F. M. Beyond the Gouy-Chapman Model with Heterodyne-Detected Second Harmonic Generation. J. Phys. Chem. Lett. 2019, 10 (10), 2328–2334. 10.1021/acs.jpclett.9b00727. 31009224

[ref65] Gonella G.; Lütgebaucks C.; de Beer A. G. F.; Roke S. Second Harmonic and Sum-Frequency Generation from Aqueous Interfaces Is Modulated by Interference. J. Phys. Chem. C 2016, 120 (17), 9165–9173. 10.1021/acs.jpcc.5b12453.

[ref66] Li H.; Xia H.; Wang D.; Tao X. Simple synthesis of monodisperse, quasi-spherical, citrate-stabilized silver nanocrystals in water. Langmuir 2013, 29, 5074–5079. 10.1021/la400214x. 23578217

[ref67] Smith H. T.; Karam T. E.; Haber L. H.; Lopata K. Capturing plasmon-molecule dynamics in dye monolayers on metal nanoparticles using classical electrodynamics with quantum embedding. J. Phys. Chem. C 2017, 121, 16932–16942. 10.1021/acs.jpcc.7b03440.

[ref68] Lopata K.; Neuhauser D. Multiscale Maxwell-Schrödinger modeling: A split field finite-difference time-domain approach to molecular nanopolaritonics. J. Chem. Phys. 2009, 130 (10), 104707 10.1063/1.3082245. 19292549

[ref69] Coomar A.; Arntsen C.; Lopata K. A.; Pistinner S.; Neuhauser D. Near-field: A finite-difference time-dependent method for simulation of electrodynamics on small scales. J. Chem. Phys. 2011, 135 (8), 084121 10.1063/1.3626549. 21895173

[ref70] Ziegler C.; Eychmuller A. Seeded growth synthesis of uniform gold nanoparticles with diameters of 15- 300 nm. J. Phys. Chem. C 2011, 115, 4502–4506. 10.1021/jp1106982.

[ref71] Zhao L.; Ji X.; Sun X.; Li J.; Yang W.; Peng X. Formation and stability of gold nanoflowers by the seeding approach: the effect of intraparticle ripening. J. Phys. Chem. C 2009, 113, 16645–16651. 10.1021/jp9058406.

[ref72] Lesuffleur A.; Kumar L. K. S.; Gordon R. Enhanced second harmonic generation from nanoscale double-hole arrays in a gold film. Appl. Phys. Lett. 2006, 88 (26), 261104 10.1063/1.2218057.

[ref73] Fukuoka N.; Tanabe K. Lightning-Rod effect of plasmonic field enhancement on hydrogen-absorbing transition metals. Nanomaterials 2019, 9 (9), 1235 10.3390/nano9091235. PMC6780797 31480329

[ref74] Butet J.; Brevet P.-F.; Martin O. J. Optical second harmonic generation in plasmonic nanostructures: from fundamental principles to advanced applications. ACS Nano 2015, 9 (11), 10545–10562. 10.1021/acsnano.5b04373. 26474346

[ref75] Gao N.; Chen Y.; Li L.; Guan Z.; Zhao T.; Zhou N.; Yuan P.; Yao S. Q.; Xu Q.-H. Shape-dependent two-photon photoluminescence of single gold nanoparticles. J. Phys. Chem. C 2014, 118 (25), 13904–13911. 10.1021/jp502038v.

[ref76] Dadap J. I.; Shan J.; Eisenthal K. B.; Heinz T. F. Second-harmonic Rayleigh scattering from a sphere of centrosymmetric material. Phys. Rev. Lett. 1999, 83 (20), 4045 10.1103/PhysRevLett.83.4045.

[ref77] Jen S.-H.; Dai H.-L.; Gonella G. The effect of particle size in second harmonic generation from the surface of spherical colloidal particles. II: The nonlinear Rayleigh- Gans- Debye model. J. Phys. Chem. C 2010, 114 (10), 4302–4308. 10.1021/jp910144c. 19278215

