# Supplementary material for: Growth Dynamics of Colloidal Silver–Gold Core–Shell Nanoparticles Studied by In Situ Second Harmonic Generation and Extinction Spectroscopy
Source: J Phys Chem C Nanomater Interfaces. 2021 Nov 15;125(46):25615–23. doi: 10.1021/acs.jpcc.1c06094 (PMC8631735; doi:10.1021/acs.jpcc.1c06094)
Supplement: Supplementary file 1 — jp1c06094_si_001.pdf [file jp1c06094_si_001.pdf]

Supporting Information for

Growth Dynamics of Colloidal Silver-Gold Core-Shell Nanoparticles Studied by *in Situ* Second Harmonic Generation and Extinction Spectroscopy

Asela S. Dikkumbura, Prakash Hamal, Min Chen, Daniel A. Babayode, Jeewan C. Ranasinghe, Kenneth Lopata,† and Louis H. Haber*

Department of Chemistry, Louisiana State University, Baton Rouge, Louisiana 70803, USA.

†Center for Computation and Technology, Louisiana State University, Baton Rouge, Louisiana 70803, USA.

*Corresponding author's email: lhaber@lsu.edu

Additional characterizations of silver and silver-gold core-shell nanoparticles

Figure S1 shows a representative transmission electron microscopy (TEM) image of silver nanoparticles and the corresponding extinction spectrum. The experimental extinction spectrum of silver nanoparticles is compared to the corresponding spectrum calculated using a finite-difference time-domain (FDTD) approach. These silver nanoparticles are used for the seed-

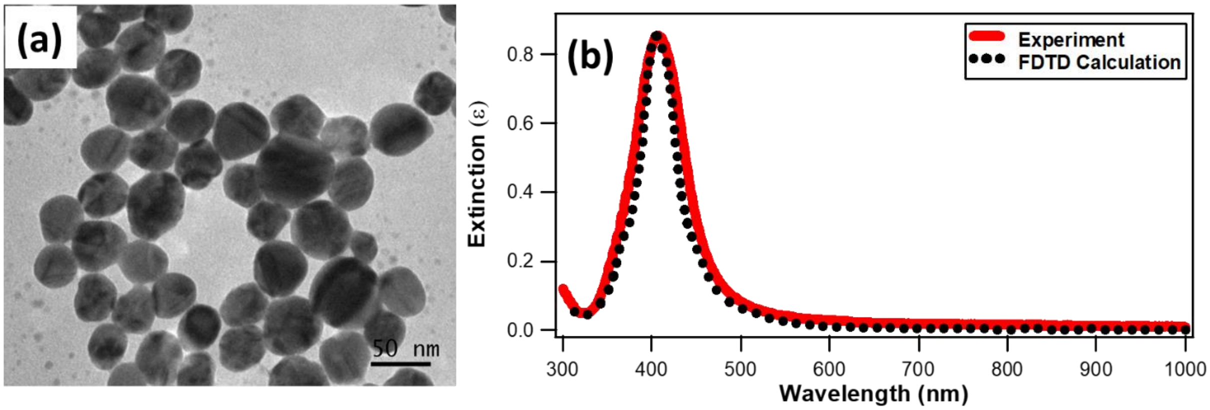

Figure S1. (a) Representative TEM image of silver nanoparticles and (b) experimental extinction spectrum of silver nanoparticles (red line) compared to corresponding spectrum calculated using an FDTD approach (dotted black line).

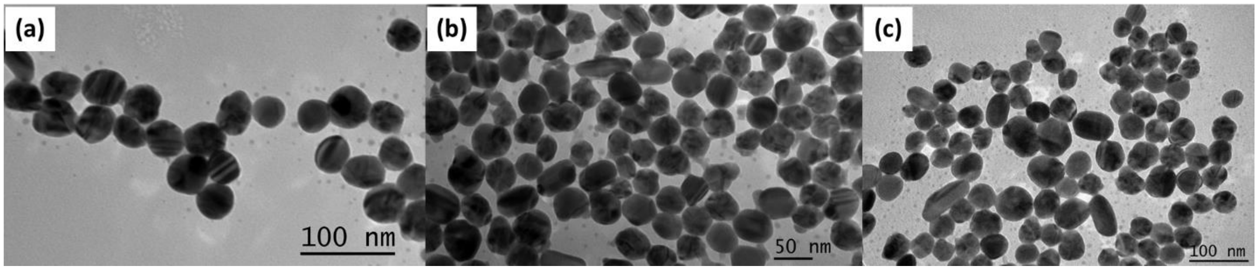

Figure S2. Additional TEM images of silver nanoparticles used for the Ag@Au CS synthesis.

mediated silver-gold core-shell (Ag@Au CS) nanoparticle synthesis, where these silver nanoparticles become the silver core. Figure S2 shows additional representative TEM images of silver nanoparticles. All TEM images are taken using a JEOL-1400 microscope with carbon-coated copper grids. Figures S3, S4, and S5 show additional representative TEM images of Ag@Au CS nanoparticles after the first, second, and third additions of $HAuCl_4$ and reducing agents, respectively, where the growth processes are monitored using *in situ* second harmonic generation (SHG) and extinction spectroscopy, as explained in the paper. All Ag@Au CS nanoparticles have a silver core of diameter 42.0 ± 6.8 nm. Figure S6 displays the nanoparticle size distribution histograms that are obtained by measuring the diameters of the nanoparticles from surveys of the TEM images. The histograms are fitted to log-normal functions, which are indicated by dotted black lines. The sizes obtained from the fits are 42.0 ± 6.8 nm for the silver nanoparticles, and 56.3 ± 7.6 nm, 94.5 ± 11.8 nm, and 114.7 ± 12.5 nm for the Ag@Au CS nanoparticles prepared using

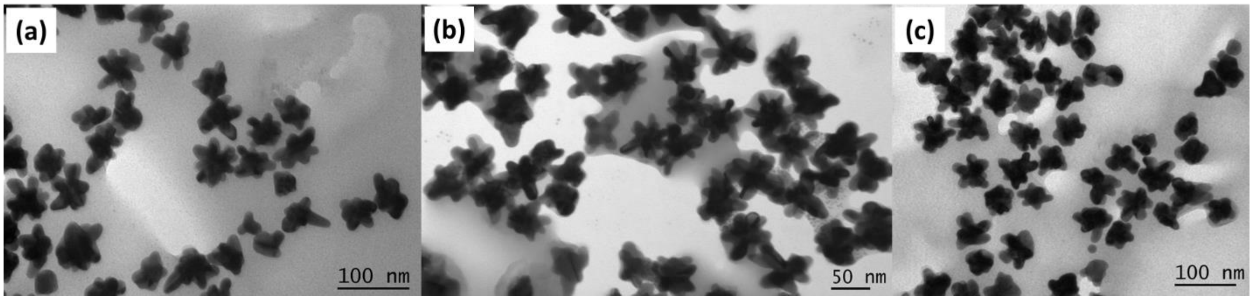

Figure S3. Additional TEM images of Ag@Au CS nanoparticles prepared after the first addition of $HAuCl_4$ and reducing agents using precursor silver nanoparticles.

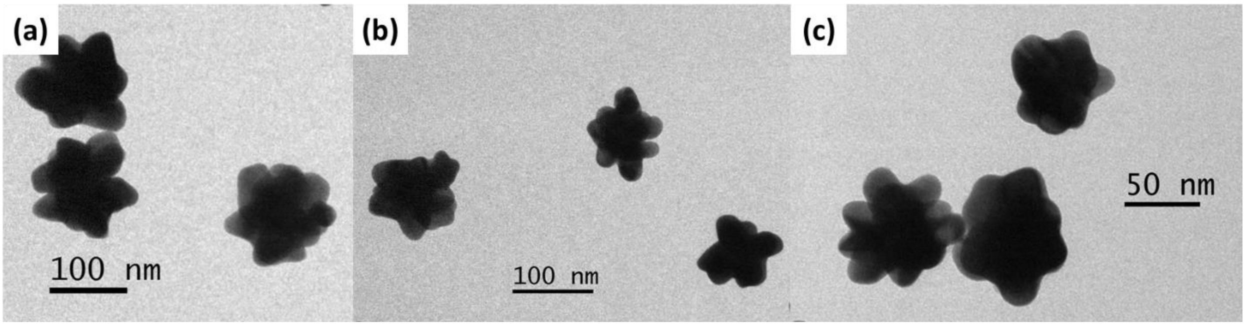

Figure S4. Additional TEM images of Ag@Au CS nanoparticles prepared after the second addition of $HAuCl_4$ and reducing agents.

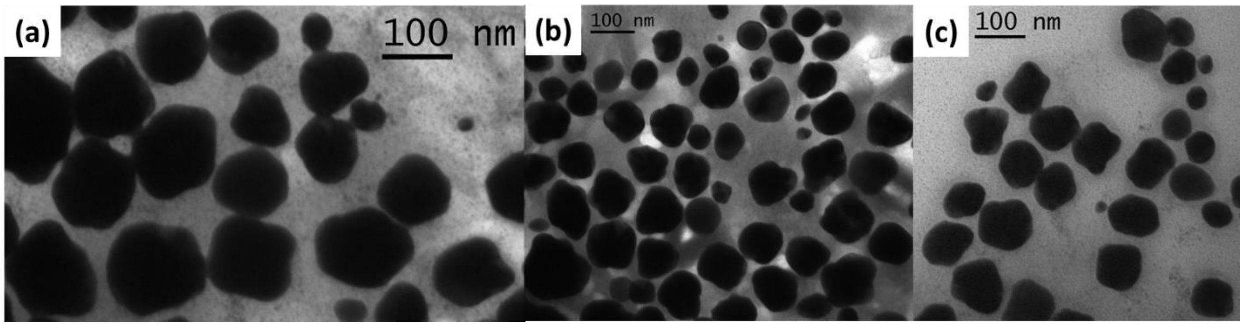

Figure S5. Additional TEM images of Ag@Au CS nanoparticles prepared after the third addition of $HAuCl_4$ and reducing agents.

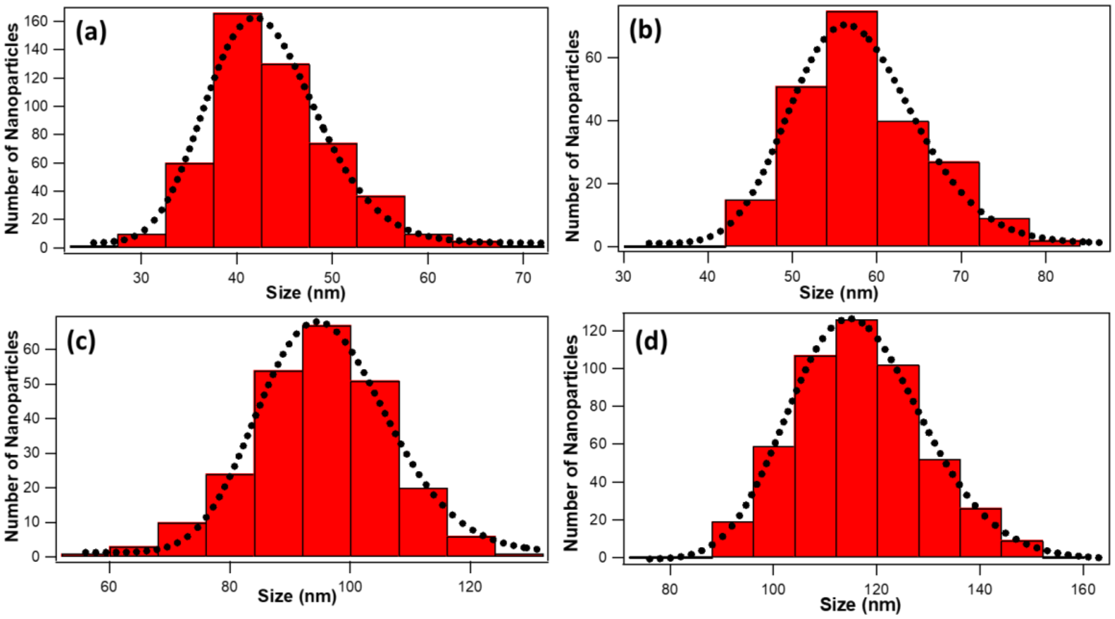

Figure S6. Size distribution histograms for (a) silver nanoparticles and Ag@Au CS nanoparticles after the (b) first, (c) second, and (d) third additions of $HAuCl_4$ and reducing agents, with corresponding log-normal fits (dotted black lines).

precursor silver nanoparticles after the first, second, and third additions of $HAuCl_4$ and reducing agents, respectively.

Additional details on *in situ* SHG and extinction spectroscopy

The *in situ* SHG and extinction spectroscopy setup[1-2] is shown in Figure S7. An optical filter is placed in front of the cuvette to remove any SHG light from the optical setup prior to the colloidal nanoparticle sample. Another filter is placed after the sample to remove the fundamental 800 nm light, while allowing the SHG signal through. The SHG signal is collected in the forward direction and refocused to the monochromator spectrograph connected to a high-sensitivity charge-coupled device (CCD) detector. This optical setup allows for the detection of the SHG signal as a function of time to capture the dynamics of the Ag@Au CS nanoparticle growth reaction. Concurrently, time-dependent *in situ* extinction spectra of Ag@Au CS nanoparticles are measured using a low-intensity broadband tungsten filament light source, which passes through the nanoparticle solution

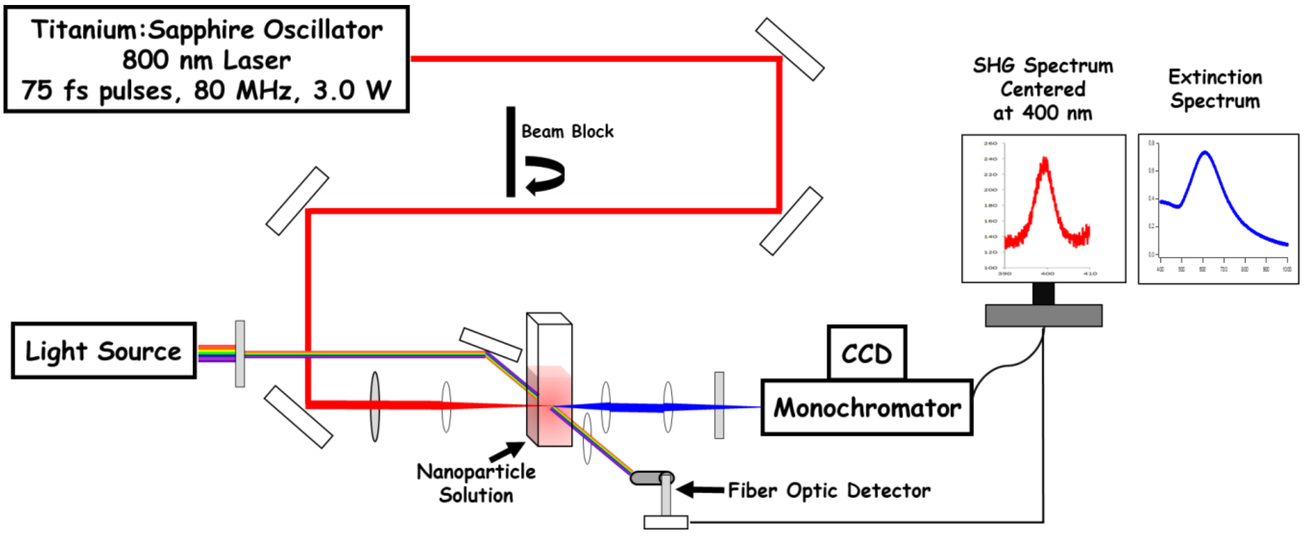

Figure S7. The *in situ* SHG and extinction spectroscopy setup.

orthogonal to the SHG beam and is then focused to a fiber optic spectrometer detector. A data acquisition program collects 5 *in situ* SHG spectra and 5 background spectra using a computer-controlled beam block, followed by 10 *in situ* extinction spectra in repeating iterations to monitor the nanoparticle core-shell growth dynamics. The SHG and extinction acquisition times are set to 1.0 s and 0.5 s, respectively.

The SHG time profiles of Ag@Au CS nanoparticles are fit using a single-exponential function to determine the SHG growth lifetimes for the three stepwise sequential additions of $HAuCl_4$ and reducing agents. Table S1 shows the tabulated SHG growth lifetimes τ_{SHG}, amplitudes A_{SHG}, and offsets B_{SHG} from the corresponding fits given by $E_{SHG}(t) = B_{SHG} + A_{SHG}e^{-\frac{t}{\tau_{SHG}}}$ with the fits shown in Figure 5. Representative *in situ* extinction spectra of Ag@Au CS nanoparticles at various times during the stepwise additions of $HAuCl_4$ and reducing agents are shown in Figure S8. The extinction peak time profiles of Ag@Au CS nanoparticles are fit using a single exponential function to determine the extinction growth lifetimes for three different additions of $HAuCl_4$ and reducing agents. The time traces of the extinction peak values and corresponding single-exponential fits during the formation of the gold shell onto the silver core are shown in Figure S9. Table S2 shows the tabulated extinction growth lifetimes τ_{ext}, amplitudes A_{ext}, and offsets B_{ext} from the corresponding fits shown in Figures 4 and S9.

Addition	τ_{SHG} (s)	B_{SHG}	A_{SHG}
1st	87 ± 6	597 ± 6	-769 ± 39
2nd	439 ± 6	613 ± 2	1987 ± 52
3rd	409 ± 13	151 ± 3	21701 ± 3030

Table S1. Fitting parameters obtained from the *in situ* SHG electric field as a function of reaction time for the three different additions.

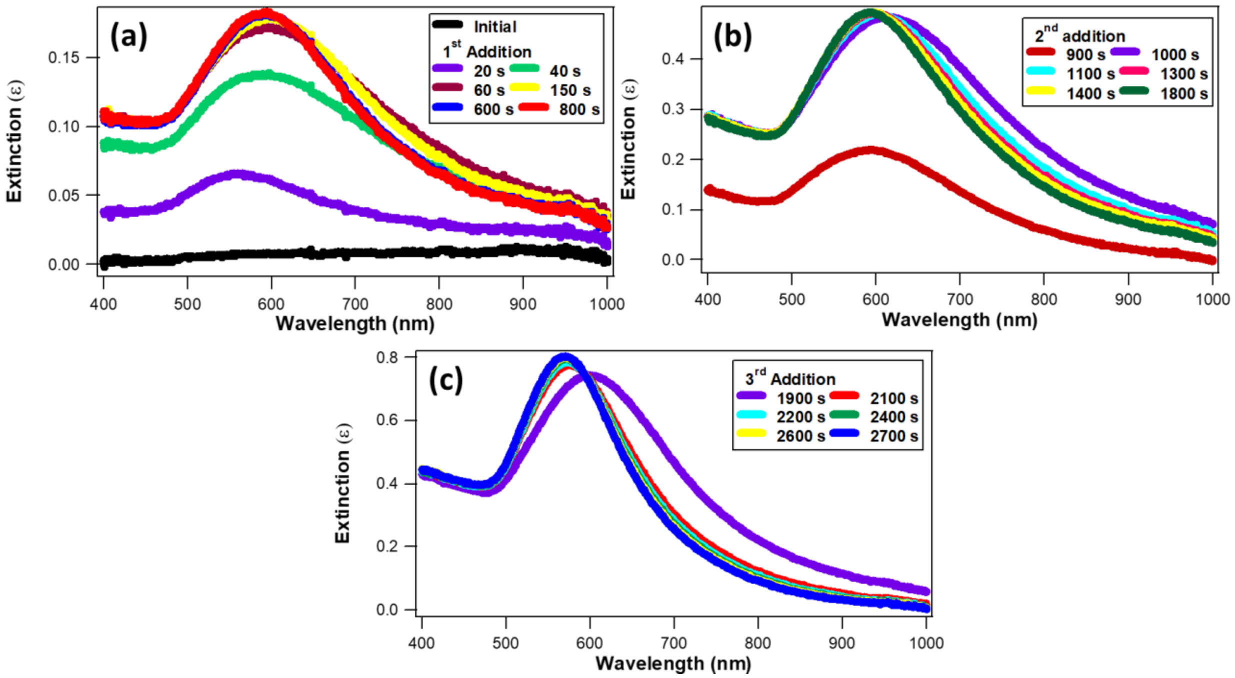

Figure S8. Representative *in situ* extinction spectra of Ag@Au CS nanoparticles during the (a) first, (b) second, and (c) third additions of $HAuCl_4$ and reducing agents, respectively, at different times during the reaction.

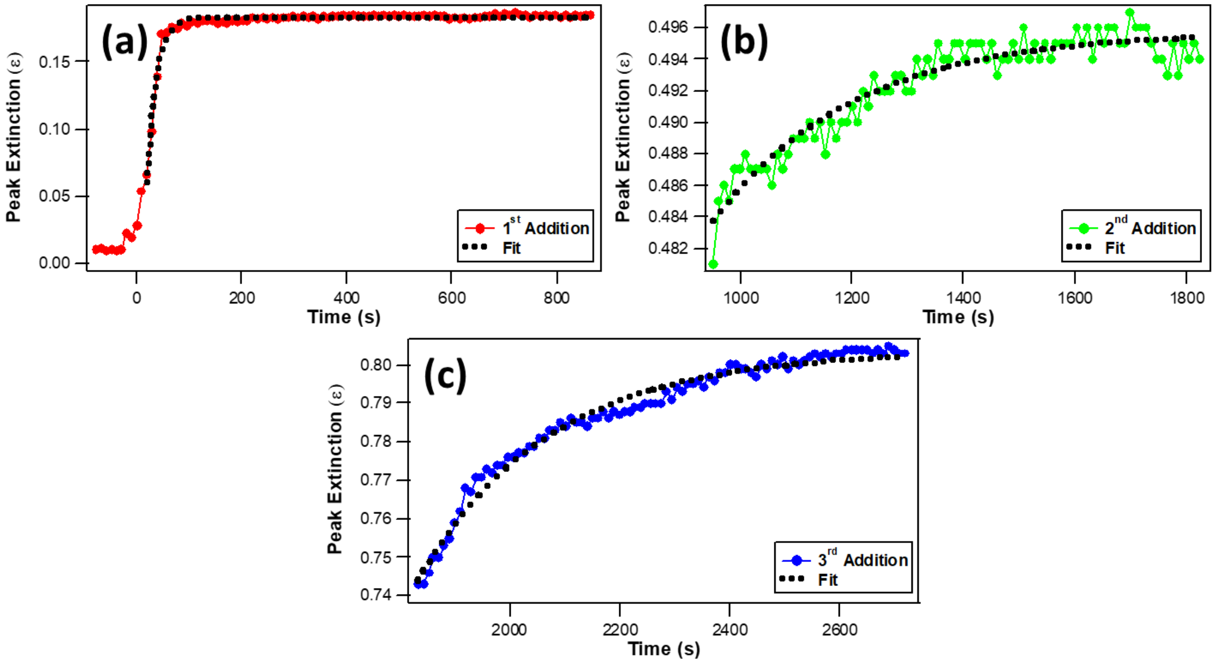

Figure S10. Extinction peak values as a function of reaction time during the Ag@Au CS nanoparticle synthesis for the (a) first (red circles), (b) second (green circles), and (c) third (blue circles) additions, respectively, compared to the corresponding fits (dotted black lines).

Addition	τ_{ext} (s)	B_{ext}	A_{ext}
1st	18 ± 1	0.1834 ± 0.0003	-0.35 ± 0.02
2nd	253 ± 24	0.4958 ± 0.0003	-0.52 ± 0.19
3rd	263 ± 11	0.8051 ± 0.0007	-0.059 ± 0.001

Table S2. Fitting parameters obtained from the *in situ* extinction peak values as a function of reaction time for the three different additions.

References

1. Ranasinghe, J. C.; Dikkumbura, A. S.; Hamal, P.; Chen, M.; Khoury, R. A.; Smith, H. T.; Lopata, K.; Haber, L. H., Monitoring the growth dynamics of colloidal gold-silver core-shell nanoparticles using in situ second harmonic generation and extinction spectroscopy. *J. Chem. Phys.* **2019,** *151*, 224701.
2. Khoury, R. A.; Ranasinghe, J. C.; Dikkumbura, A. S.; Hamal, P.; Kumal, R. R.; Karam, T. E.; Smith, H. T.; Haber, L. H., Monitoring the Seed-Mediated Growth of Gold Nanoparticles Using in Situ Second Harmonic Generation and Extinction Spectroscopy. *J. Phys. Chem. C* **2018,** *122*, 24400-24406.
